# Quantitative determination and pattern recognition analyses of bioactive marker compounds from Dipsaci Radix by HPLC

**DOI:** 10.1007/s12272-013-0162-y

**Published:** 2013-07-23

**Authors:** Bing Tian Zhao, Su Yang Jeong, Dong Cheul Moon, Kun Ho Son, Jong Keun Son, Mi Hee Woo

**Affiliations:** 1 College of Pharmacy, Catholic University of Daegu, Gyeongsan, 712-702 Korea; 2 College of Pharmacy, Chungbuk National University, Cheongju, Korea; 3 College of Life Science, Andong National University, Andong, Korea; 4 College of Pharmacy, Yeungnam University, Gyeongsan, 712-749 Korea

**Keywords:** Dipsaci Radix, *Dipsacus asperoides*, HPLC–UV, Quality control

## Abstract

In this study, quantitative and pattern recognition analyses were developed using HPLC/UV for the quality evaluation of Dipsaci Radix. For quantitative analysis, five major bioactive compounds were assessed. The separation conditions employed for HPLC/UV were optimized using ODS C_18_ column (250 × 4.6 mm, 5 μm) with a gradient of acetonitrile and water as the mobile phase at a flow rate of 1.0 mL/min and a detection wavelength of 212 nm. These methods were fully validated with respect to linearity, accuracy, precision, recovery, and robustness. The HPLC/UV method was applied successfully to the quantification of five major compounds in the extract of Dipsaci Radix. The HPLC analytical method for pattern recognition analysis was validated by repeated analysis of 17 Dipsaci Radix and four Phlomidis Radix samples. The results indicate that the established HPLC/UV method is suitable for quantitative analysis.

## Introduction

The use of herbal medicines continues to expand rapidly throughout the world. Many people now take herbal medicines or herbal products for their health in different national health-care settings (WHO 2004). The requirements and methods for quality control of finished herbal products, particularly for mixed herbal products, are far more complex than for other pharmaceuticals. The quality of such products is influenced by the quality of the raw materials used. Good agricultural and good collection practices (GACP) for medicinal plants, including plant selection and cultivation, are therefore important measures (WHO 2003). Quality control in synthetic drugs is conducted by measuring their medicinal components, whereas quality control in herbal medicines is traditionally performed by measuring a representative compound (a marker compound) contained in the herbal medicines. However, quantitation of one or a few components is not an adequate approach for quality control of herbal medicines. Thus there is an urgent need to establish a comprehensive qualified evaluation method based on analysis of the bioactive compounds in order to accurately reflect the quality of herbal medicines. Fingerprint analysis/pattern recognition with multivariate statistical analysis can provide information regarding the overall chemical composition of herbal medicines, including the marker compounds traditionally used for quality control (Islam et al. 2009).

Dipsaci Radix is the roots of *Dipsacus asperoides* C. Y. Cheng et T. M. Ai (Dipsacaceae) in the Korean Herbal Pharmacopoeia (K. H. P.) (Korea Food and Drug Administration 2007) and the Chinese Pharmacopoeia (C. P.). Dipsaci Radix is controlled to contain >2 % of akebia saponin D in C. P. (Pharmacopoeia Commission of People’s Republic of China 2010). Various saponins (Hung et al. 2005; Oh et al. 1999), iridoids (Tomita and Mouri 1996) and phenylpropanoids (Inoue et al. 1989) have been isolated from Dipsaci Radix. Biological studies have revealed that this plant possesses antioxidant (Hung et al. 2006), anti-inflammatory (Jung et al. 2012), anticomplementary (Oh et al. 1999), and cytotoxic effects (Tomita and Mouri 1996; Zhou et al. 2009).

Dipsaci Radix and Phlomidis Radix are different species. Phlomidis Radix is the roots of *Phlomis umbrosa* Turczaninow in the Korean Herbal Pharmacopoeia (K. H. P.) (Korea Food and Drug Administration 2007). However, Phlomidis Radix has often been misused as Dipsaci Radix in the Korean market because of similarities in their names and shapes. Therefore, we have selected Phlomidis Radix as a comparative herbal medicine. There is no report on the differentiation between Dipsaci Radix and Phlomidis Radix.

Some HPLC/UV analytical methods have been developed for the analysis of Dipsaci Radix and its related products. Ma et al. (2007) and Wang et al. (2006) reported akebia saponin D as a specific marker for the distinction of Dipsaci Radix. Those studies focused only on quantitative analysis of akebia saponin D, which is not a promising approach for the quality control or fingerprinting analysis of herbal drugs. However, as multiple compounds might be associated with therapeutic functions, a single marker compound could not be responsible for the overall pharmacological activities of Dipsaci Radix. Therefore, there is an urgent need to establish a comprehensive quality evaluation method based on analysis of a variety of active compounds in order to accurately reflect the quality of these herbal drugs. In the present study, a simple, sensitive and precise reverse-phase HPLC/UV method has been developed for the quantitative determination of five marker components, loganin (**1**), sweroside (**2**), dipsanoside A (**3**), 3-*O*-[*β*-d-glu-(1→4)][*α*-l-rha-(1→3)]-*β*-d-glu(1→3)-*α*-l-rha-(1→2)-*α*-l-ara-hed 28-*O*-*β*-d-glu-(1→6)-*β*-d-glu ester (**4**) and akebia saponin D (**5**), along with a pattern-recognition method for the quality control of Dipsaci Radix extract. Using this method, the contents of bioactive compounds in seventeen Dipsaci Radix and four Phlomidis Radix samples from China and Korea were analyzed and compared.

Using this method, the contents of bioactive compounds in seventeen Dipsaci Radix from China and four Phlomidis Radix from Korea samples were analyzed and compared. We used all Dipsaci Radix samples from China, because its Korean samples couldn’t be available in the herbal markets. The PAM method of pattern analysis was subsequently applied to the quality control of the roots of Dipsaci Radix and Phlomidis Radix.

## Materials and methods

### Plant materials

Twenty-one samples including seventeen Dipsaci Radix and four Phlomidis Radix samples cultivated in different regions were provided by the National Center for Standardization of Herbal Medicine. Dipsaci Radix (D01–D17) and Phlomidis Radix (P18–P21) included D01 (Si Chuan Sheng, China), D02 (Ji Zhou, China), D03 (unknown area, China), D04 (unknown area, China), D05 (unknown area, China), D06 (unknown area), D07 (unknown area), D08 (unknown area), D09 (unknown area, China), D10 (unknown area), D11 (An Guo, China), D12 (Si Chuan Sheng, China, processed with salt-water), D13 (unknown area, processed with salt-water), D14 (unknown area, processed with wine), D15 (Si Chuan Sheng, China, processed with wine), D16 (Nan Zhou, China), D17 (unknown area, China), P18 (Yeong Cheon, Korea), P19 (Je Chon, Korea), P20 (Yeong Cheon, Korea) and P21 (Yeong Cheon, Korea).

### Reagents

**Fig. 1:**
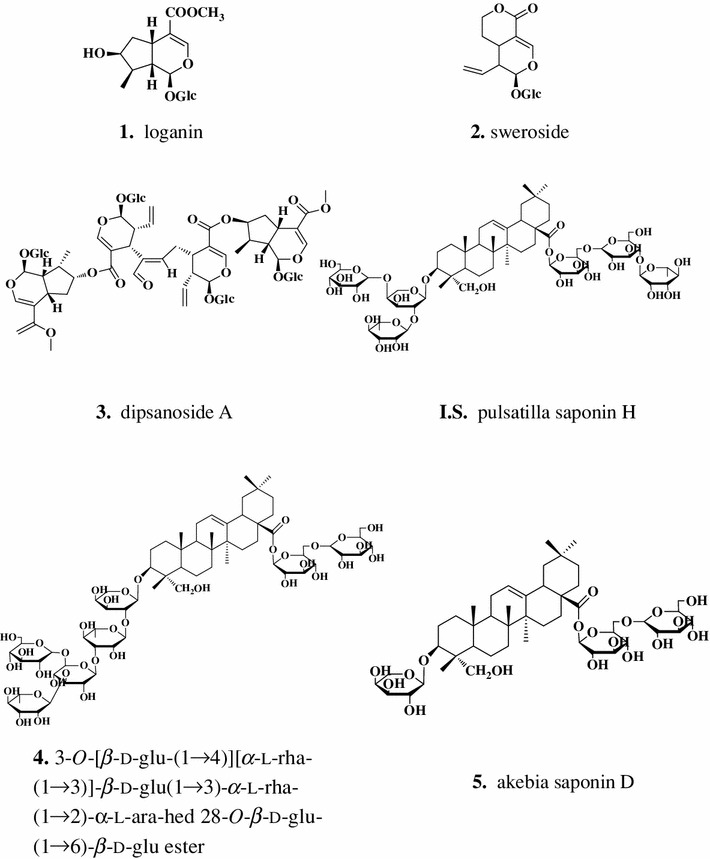
Chemical structures of standards

All of the standard compounds were provided by Prof. Kun Ho Son, Andong National University, Andong, Korea. Their structures were unambiguously identified as loganin (**1**), sweroside (**2**), dipsanoside A (**3**), 3-*O*-[*β*-d-glu-(1→4)][*α*-l-rha-(1→3)]-*β*-d-glu(1→3)-*α*-l-rha-(1→2)-*α*-l-ara-hed 28-*O*-*β*-d-glu-(1→6)-*β*-d-glu ester (**4**) and akebia saponin D (**5**) based on NMR and MS data compared with published data. The standard compound structures are shown in Fig. 1. Purity of standard compounds was estimated to be higher than 95 % based on HPLC and LC–MS/MS analysis. Internal standard (**I**.**S**.), pulsatilla saponin H, was provided as powder from Prof. Sam Sik Kang, Seoul National University. HPLC-grade methanol and acetonitrile were purchased from Merck K GaA (Darmstadt, Germany). All other chemicals used were of analytical grade unless otherwise noted. Distilled water was prepared using Milli-Q purification system (Millipore, Bedford, MA, USA).

### Sample preparation

Dried rhizome powder was used to determine the contents of the five marker compounds and pattern recognition analysis of each extraction of Dipsaci Radix. Powdered Dipsaci Radix was sieved through 50 mesh, and about 0.5 g of the powder was accurately weighed; 25 mL of 50 % methanol were added, the weight was accurately measured, and the sample was sonicated for 30 min. The solution was weighed again, and the loss in weight was made up with 50 % methanol. The solution was filtered through a 0.45-μm membrane filter and the filtrate was used as the test solution. Sample solution of 20 μL was injected into the HPLC system.

### HPLC/UV conditions

The HPLC equipment was a Waters HPLC system (Waters, Milford, MA, USA) with a Waters 600 pumps, a Waters 486 UV detector and a Waters 717 autosampler. YMC ODS-H80 (250 × 4.6 mm, 4 μm), Shiseido capcell pak (250 × 4.6 mm, 5 μm) and Shodex ODS pak (250 × 4.6 mm, 5 μm) columns were tested with the guard column filled with the same stationary phase. A (100 % acetonitrile) and B (water) were used as the mobile phase under a gradient condition (0 min, 15 % A; 60 min, 35 % A). The mobile phase was filtered under vacuum through a 0.45-μm membrane filter and degassed prior to use. The analysis was carried out at a flow rate of 1.0 mL/min with the detection wavelength set to 212 nm, and the total run time was 60 min. All compounds could be resolved with baseline separation at 212 nm with maximum absorption. Hence, characteristic chromatographic patterns were obtained at 212 nm. The chromatograms were processed using Empower Pro software, Build 1154 (Waters, Milford, MA).

### Analytical method validation

The standards (4 mg) of loganin (**1**), sweroside (**2**), dipsanoside A (**3**), 3-*O*-[*β*-d-glu-(1→4)][*α*-l-rha-(1→3)]-*β*-d-glu(1→3)-*α*-l-rha-(1→2)-*α*-l-ara-hed 28-*O*-*β*-d-glu-(1→6)-*β*-d-glu ester (**4**) and akebia saponin D (**5**) were each accurately weighed and then dissolved in 10 mL of 100 % methanol to produce stock standard solutions of 400 ppm. The internal standard (pulsatilla saponin H; 15 mg) was accurately weighed and then dissolved in 10 mL of 100 % methanol to produce a stock solution of 1,500 ppm. The calibration curves were made by diluting the stock solutions with 100 % methanol. A reference solution of the five standard compounds at concentrations of 0.1–200 μg/mL was analyzed by HPLC/UV. The regression equations were calculated in the form of *y* = *ax* + *b*, where *y* and *x* correspond to peak area ratio for internal standard and compound concentration, respectively. Recovery tests were executed by mixing a powdered sample (0.5 g) with the reference compounds at three control levels (near the LOQ, medium and higher concentrations for calibration curve of each compound contained in the samples). The mixture was then extracted by sonication in 25 mL of 50 % methanol for 30 min. The extract solution was filtered through a 0.45-μm membrane. The HPLC/UV analysis experiments were performed in triplicate for each control level. The data from the standard solution and the extracted sample were compared. Precision and accuracy were determined by multiple analyses (*n* = 5) of quality control samples prepared at low, medium and high concentrations spanning the calibration range.

### Pattern recognition analysis

To evaluate the phytochemical equivalency among the 21 samples comprising seventeen Dipsaci Radix and four Phlomidis Radix samples, pattern recognition analysis was conducted. In this study we used three marker compound peaks [sweroside (**2**), dipsanoside A (**3**) and akebia saponin D (**5**)] for pattern recognition analysis. Pattern recognition analysis was conducted using software package R-2.11.0.

## Results and discussion

### Optimization of chromatographic conditions

**Fig. 2:**
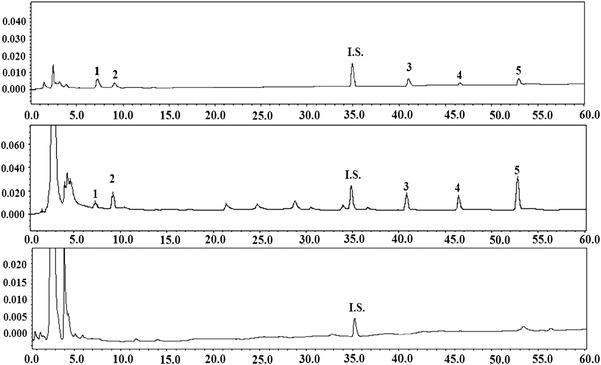
HPLC chromatograms of standard mixture (**a**), Dipsaci Radix (**b**) and Phlomidis Radix (**c**). **1** loganin, **2** sweroside, **3** dipsanoside A, **4** 3-*O*-[*β*-d-glu-(1→4)][*α*-l-rha-(1→3)]-*β*-d-glu(1→3)-*α*-l-rha-(1→2)-α-l-ara-hed 28-*O*-*β*-d-glu-(1→6)-*β*-d-glu ester, **5** akebia saponin D, **I.S.** pulsatilla saponin H

The HPLC conditions were selected according to the requirement for obtaining chromatograms with better resolution of adjacent peaks within a short retention time. For the optimization of chromatographic conditions, the effect of the mobile phase composition on the separation was examined. A mobile phase of water–methanol did not result in satisfactory separation of structurally similar compounds. Acetonitrile as an organic modifier significantly improved the separation. We also tested the addition of 0.1, 1 and 10 % acid (acetic acid, formic acid and phosphoric acid) in the water. The water without acid resulted in good resolution of all compounds, as well as satisfactory peak symmetry and shape. The typical chromatograms of samples and standard mixture are shown in Fig. 2, which shows that all target compounds and an internal standard are completely separated within 60 min. Pulsatilla saponin H (**I.S.**) was selected as an internal standard. The chromatographic peaks of the analytes in sample solution were identified by comparing their retention times with those of the reference standards and were further confirmed by spiking samples with reference compounds (Fig. 2). All compounds could be resolved with baseline separation at 212 nm with the maximum absorption shown for the five constituents. Hence, characteristic chromatographic patterns were obtained at 212 nm.

### Optimization of sample preparation conditions

Eight extracting solvents, 100 % ethanol, 75 % ethanol, 50 % ethanol, 25 % ethanol, 100 % methanol, 75 % methanol, 50 % methanol and 25 % methanol, were compared in sample assays after sonication for 30 min. When the sample was extracted with 50 % methanol, the sample assay was higher than the other solvent samples. Therefore, we employed 50 % methanol as the extracting solvent throughout this work. Two extraction methods, ultra-sonication and reflux using 50 % methanol as an extraction solvent, were compared in sample assays. The sample assay results after the sonication extraction method were higher than those after reflux. To determine the time needed for complete extraction, samples were extracted for five different lengths of time (10, 20, 30, 40 and 60 min). Thus 50 % methanol solvent and the sonication extraction method were employed. When the extraction time was 30 min, the sample assay results were similar to those at 40 min. Therefore, when the extraction time was 30 min, all of the compounds were sufficiently extracted.

### Validation of the method

#### Linearity, calibration range, limits of detection and quantification

Each coefficient of correlation (*r* ^2^) was > 0.999, as determined by least square analysis, suggesting good linearity between the peak area ratio and the compound concentrations (Table 1). The limits of detection (LOD) and limits of quantitation (LOQ) were evaluated based on the lowest detectable peak in the chromatogram having a signal-to-noise (S/N) ratio of 3 and 10, respectively. The LOD and LOQ under our experimental conditions are listed in Table 1. The obtained values for both LOD and LOQ for these five standards were low enough to detect traces of these compounds in either crude extract or its preparation.

**Table 1:** Linearity, linear ranges, LOD and LOQ

Analytes	Linear range (μg/mL)	Slope (a)	Intercept (b)	Correlation coefficient (*r* ^*2*^)	LOD (ng/mL)	LOQ (ng/mL)
**1**	0.1–40	0.079	0.0318	0.9998	120	350
**2**	0.1–40	0.054	−0.0120	0.9999	140	320
**3**	0.1–50	0.1745	−0.0057	0.9999	100	290
**4**	0.1–100	0.0389	−0.0508	0.9998	130	270
**5**	0.1–200	0.0376	−0.0522	0.9999	120	330

**1** loganin, **2** sweroside, **3** dipsanoside A, **4** 3-*O*-[*β*-d-glu-(1→4)][*α*-l-rha-(1→3)]-*β*-d-glu(1→3)-*α*-l-rha-(1→2)-α-l-ara-hed 28-*O*-*β*-d-glu-(1→6)-*β*-d-glu ester, **5** akebia saponin D

#### Precision and accuracy

The extraction recovery test was performed by extracting a known amount of the five compounds from the Dipsaci Radix powder samples. Known amounts of each standard compound at three levels were mixed with the sample powder and extracted with 50 % methanol, as described in the experimental section. The % recovery of each standard ranged from 97.98 to 105.12 %, and the RSD was <1.44 % (Table 2). The average recovery was represented by the formula: R (%) = [(amount from the sample spiked standard − amount from the sample)/amount from the spiked standard] × 100.

**Table 2:** Recoveries of marker compounds through standard addition (n = 6)

Analytes	Fortified conc. (μg/mL)	Observed conc. (μg/mL)	RSD^a^ (%)	Calculated recovery (%)	Mean recovery (%)
**1**	0.0	8.47	–	–	105.12
	0.4	8.92	0.30	112.50	
	20.0	28.89	0.38	102.10	
	40.0	48.77	0.06	100.75	
**2**	0.0	51.29	–	–	99.35
	0.4	51.26	0.20	92.50	
	20.0	72.15	0.11	104.30	
	40.0	91.79	0.04	101.25	
**3**	0.0	17.38	–	–	101.49
	0.3	17.69	0.50	103.33	
	20.0	37.67	0.04	101.45	
	50.0	67.22	0.02	99.68	
**4**	0.0	80.13	–	–	97.98
	0.3	80.42	0.04	96.67	
	50.0	128.20	0.02	96.14	
	100.0	181.26	0.01	101.13	
**5**	0.0	111.27	–	–	101.72
	0.3	111.58	0.07	103.33	
	100.0	211.5	0.01	100.23	
	200.0	314.48	0.01	101.61	

**1** loganin, **2** sweroside, **3** dipsanoside A, **4** 3-*O*-[*β*-d-glu-(1→4)][*α*-l-rha-(1→3)]-*β*-d-glu(1→3)-*α*-l-rha-(1→2)-α-l-ara-hed 28-*O*-*β*-d-glu-(1→6)-*β*-d-glu ester, **5** akebia saponin D

^a^Relative standard deviation

Intra-assay precision and accuracy were determined from the variability of multiple analyses (*n* = 5) of quality control samples analyzed within the same analytical run. The quality control samples had intra-assay precision below 0.50 % and accuracy between 96.67 and 102.49 %. Inter-assay precision and accuracy were evaluated from the variability of multiple analyses (*n* = 5) of quality control samples analyzed on a single analytical run for consecutive 5 days. The quality control samples had an inter-assay precision of <2.42 % and accuracy between 97.50 and 103.33 %. Thus, the developed method is highly reproducible; the precision and accuracy data are presented in Table 3.

**Table 3:** Precision and accuracy of analytical results (n = 5)

Analytes	Nominal conc.^a^ (μg/mL)	Intra-day (*n* = 5)				Inter-day (n = 5)			
		Observed^b^ (μg/mL)	S.D.^c^	Accuracy^d^ (%)	Precision^e^	Observed (μg/mL)	S.D.	Accuracy (%)	Precision
**1**	0.4	0.41	0.01	102.49	0.30	0.41	0.02	102.50	1.06
	20.0	20.09	0.01	100.45	0.38	20.03	0.08	100.15	1.62
	40.0	40.04	0.01	100.10	0.06	40.05	0.14	100.13	1.40
**2**	0.4	0.39	0.02	97.50	0.20	0.39	0.06	97.50	0.58
	20.0	19.78	0.02	98.91	0.11	20.12	0.47	100.60	2.42
	40.0	40.01	0.02	100.02	0.04	40.19	0.91	100.48	2.34
**3**	0.3	0.31	0.02	96.67	0.50	0.31	0.10	103.33	2.08
	20.0	20.07	0.03	100.34	0.04	20.02	0.13	100.10	1.26
	50.0	49.78	0.01	99.57	0.02	50.06	0.36	100.12	1.79
**4**	0.3	0.29	0.01	96.67	0.04	0.30	0.42	100.00	2.11
	50.0	49.72	0.02	99.44	0.02	49.98	0.49	99.96	1.21
	100.0	99.92	0.02	99.92	0.01	100.36	0.93	100.36	1.71
**5**	0.3	0.29	0.02	96.67	0.07	0.30	0.35	100.00	1.40
	100.0	99.88	0.01	99.88	0.01	100.04	0.55	100.04	1.10
	200.0	200.09	0.01	100.05	0.01	200.12	0.61	100.06	0.62

^a^Added concentration of standard

^b^Amount from the sample spiked standard − amount from the sample

^c^Standard deviation

^d^(Observed/added) × 100

^e^The relative standard deviation of accuracy

**1** loganin, **2** sweroside, **3** dipsanoside A, **4** 3-*O*-[*β*-d-glu-(1→4)][*α*-l-rha-(1→3)]-*β*-d-glu(1→3)-*α*-l-rha-(1→2)-α-l-ara-hed 28-*O*-*β*-d-glu-(1→6)-*β*-d-glu ester, **5** akebia saponin D

#### Robustness

The robustness was determined in order to evaluate the reliability of the established HPLC method. All of the parameters were maintained so there would not be any interference with other peaks for the Dipsaci Radix. The experimental conditions, such as column temperature, column species and flow rate, were purposely altered. The theoretical plate (*N*), capacity factor (*k′*), separation factor (*α*) and resolution (*R*s) were evaluated. To evaluate their suitability, three different columns, YMC, Phenomenex and Shodex, were compared with regard to four analytical factors (*N*, *k′*, *α* and *R*s) on the column temperature of 30 °C. The result showed that the four analytical factors did not differ greatly among the column species. Four different column temperatures, 25, 30, 35 and 40 °C, were compared with regard to these four analytical factors using the YMC column. Again the four analytical factors did not differ greatly by column temperature. Three different flow rates, 0.9, 1.0 and 1.1 mL/min, were also compared with regard to the four analytical factors using the YMC column at 30 °C. The four analytical factors did not differ greatly by flow rate. We sought to optimize the chromatographic parameters, but the four analytical factors did not differ greatly when the conditions were changed; therefore these experimental conditions were sufficiently robust.

The sample stability was tested with a standard mixture solution at 0, 0.5, 1, 2, 5, 10, 15 and 30 days. During this period, the solution was stored in the dark at room temperature or at 4 °C. The resulting data indicated that all marker analytes remained stable (99.99 %) during the experimental period.

#### Sample analysis

The developed HPLC/UV method was then applied to the simultaneous determination of the five compounds, loganin (**1**), sweroside (**2**), dipsanoside A (**3**), 3-*O*-[*β*-d-glu-(1→4)][*α*-l-rha-(1→3)]-*β*-d-glu(1→3)-*α*-l-rha-(1→2)-*α*-l-ara-hed 28-*O*-*β*-d-glu-(1→6)-*β*-d-glu ester (**4**) and akebia saponin D (**5**) in Dipsaci Radix and Phlomidis Radix. The quantity of each compound present in samples was determined and the results are summarized in Table 4. Each sample was analyzed in triplicate to ensure the reproducibility of the quantitative result. Loganin (0.01–0.38 %), sweroside (0.28–0.98 %), dipsanoside A (0.01–0.39 %), 3-*O*-[*β*-d-glu-(1→4)][*α*-l-rha-(1→3)]-*β*-d-glu(1→3)-*α*-l-rha-(1→2)-*α*-l-ara-hed 28-*O*-*β*-d-glu-(1→6)-*β*-d-glu ester (0.01–1.70 %) and akebia saponin D (0.73–10.96 %) were found in Dipsaci Radix. These Dipsaci Radix components clustered into one group, and the most abundant component was akebia saponin D (0.73–10.96 %). Contents of akebia saponin D in D12 and D13 for salt-water processing were 1.70 and 0.73 %, respectively. Neither D12 nor D13 was suitable based on the regulation of >2 % in the Chinese Pharmacopoeia [5]. Thus, the major compounds in Dipsaci Radix appeared to change due to salt-water processing. In contrast, compounds **1**–**5** were not completely contained in Phlomidis Radix as a comparison herbal medicine. In the quantitative analysis, Dipsaci Radix and Phlomidis Radix samples clustered into two groups as mentioned below.

**Table 4:** Contents (wt%) of five components in Dipsaci Radix (D01–D17) and Phlomidis Radix (P18–P21) samples

Sample	Contents (w/w %)				
	**1**	**2**	**3**	**4**	**5**
D01	0.06 ± 0.01^a^	0.98 ± 0.01	0.29 ± 0.01	1.67 ± 0.06	10.62 ± 0.52
D02	0.01 ± 0.00	0.28 ± 0.00	0.15 ± 0.01	1.60 ± 0.06	3.61 ± 0.09
D03	0.22 ± 0.01	0.87 ± 0.02	0.28 ± 0.01	0.88 ± 0.01	10.81 ± 0.29
D04	0.08 ± 0.01	0.75 ± 0.00	0.24 ± 0.01	1.70 ± 0.03	10.96 ± 0.10
D05	0.01 ± 0.00	0.52 ± 0.02	0.39 ± 0.01	1.05 ± 0.02	6.41 ± 0.16
D06	0.05 ± 0.01	0.73 ± 0.03	0.24 ± 0.02	1.53 ± 0.02	8.14 ± 0.13
D07	0.07 ± 0.01	0.42 ± 0.01	0.27 ± 0.01	0.83 ± 0.03	4.80 ± 0.05
D08	0.04 ± 0.01	0.40 ± 0.02	0.20 ± 0.01	0.84 ± 0.01	5.05 ± 0.06
D09	0.05 ± 0.01	0.73 ± 0.01	0.35 ± 0.01	1.26 ± 0.03	9.18 ± 0.30
D10	0.07 ± 0.01	0.70 ± 0.01	0.36 ± 0.01	1.21 ± 0.02	4.00 ± 0.16
D11	0.11 ± 0.01	0.28 ± 0.01	0.14 ± 0.00	0.44 ± 0.01	3.71 ± 0.10
D12	0.06 ± 0.01	0.64 ± 0.02	0.28 ± 0.00	1.28 ± 0.04	1.70 ± 0.01
D13	0.16 ± 0.01	0.38 ± 0.00	0.01 ± 0.00	0.01 ± 0.00	0.73 ± 0.02
D14	0.38 ± 0.01	0.70 ± 0.02	0.08 ± 0.01	0.44 ± 0.02	3.16 ± 0.06
D15	0.15 ± 0.01	0.87 ± 0.03	0.13 ± 0.01	0.55 ± 0.01	9.16 ± 0.14
D16	0.05 ± 0.01	0.55 ± 0.02	0.22 ± 0.01	1.36 ± 0.06	7.30 ± 0.24
D17	0.01 ± 0.00	0.32 ± 0.00	0.18 ± 0.01	0.98 ± 0.03	5.45 ± 0.15
Average^b^	0.09 ± 0.00	0.60 ± 0.01	0.22 ± 0.01	1.04 ± 0.04	6.16 ± 0.12
P18	–	–	–	–	–
P19	–	–	–	–	–
P20	–	–	–	–	–
P21	–	–	–	–	–

^a^Each value represents the mean ± S.D. (*n* = 3)

^b^The average contents of all the Dipsaci Radix (D01–D17)

#### Pattern recognition analysis

**Fig. 3:**
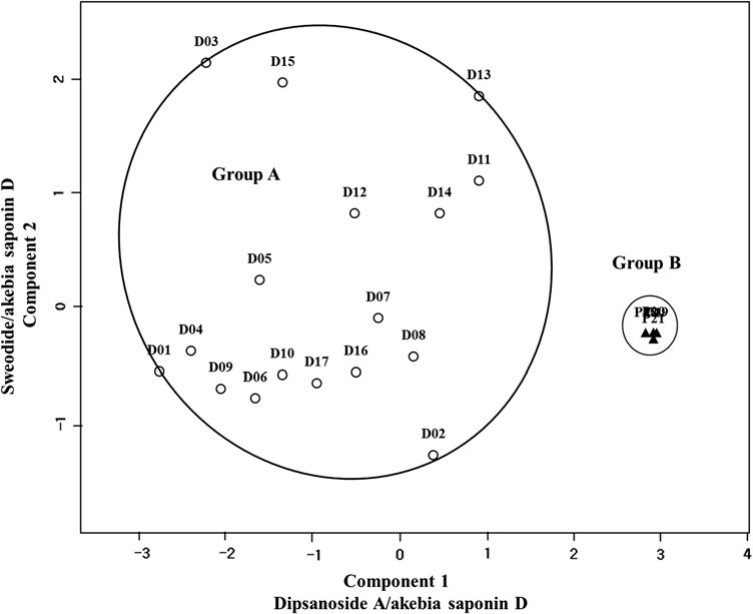
PAM of Dipsaci Radix (A: D01–D17) and Phlomidis Radix (B: P18–P21)

To evaluate the phytochemical equivalency between 17 Dipsaci Radix and four Phlomidis Radix samples, pattern recognition analysis was conducted. In this study we used three marker compound peaks [sweroside (**2**), dipsanoside A (**3**) and akebia saponin D (**5**)] for pattern recognition analysis. Even though the content of compound **4** in Dipsaci Radix was higher than those of compounds **2** and **3**, we selected **2**, **3** and **5** as marker compounds rather than **4** because of difficulties in the isolation and the availability of **4**. From the pattern analysis of Partitioning Around Medoids (PAM) analyses (Fig. 3), all of the samples were clustered into two groups: A (D01–D17, Dipsaci Radix) and B (P18–P21, Phlomidis Radix).
